# How to Train Your Fungus

**DOI:** 10.1128/mBio.03031-19

**Published:** 2019-12-17

**Authors:** John G. Gibbons

**Affiliations:** a Department of Food Science, University of Massachusetts, Amherst, Massachusetts, USA; b Molecular and Cellular Biology Graduate Program, University of Massachusetts, Amherst, Massachusetts, USA; c Organismic and Evolutionary Biology Graduate Program, University of Massachusetts, Amherst, Massachusetts, USA

**Keywords:** experimental evolution, food microbiology, mycology

## Abstract

Domestication led to profound changes in human culture. During this period, humans used breeding strategies to select for desirable traits in crops and livestock. These practices led to genetic and phenotypic changes that are trackable through archaeological and genomic records. Bacteria, yeasts, and molds also experienced domestication during the agricultural revolution, but the effects of domestication on microbes are poorly understood in comparison to plants and animals. Bodinaku et al. used experimental evolution to track the phenotypic changes that occur when wild *Penicillium* molds specialize and adapt to the cheese environment (I. Bodinaku, J. Shaffer, A. B. Connors, J. L. Steenwyk, et al., mBio 10:e02445-19, 2019, https://mbio.asm.org/content/10/5/e02445-19.long). Amazingly, after only eight generations of growth in a laboratory cheese environment, mutants emerged whose traits resembled those of the Brie and Camembert cheese mold Penicillium camemberti. This study demonstrated that the early stages of microbial domestication can occur rapidly and suggested that experimental evolution may be a viable strategy to exploit the metabolic diversity of wild microbes for food fermentation.

## Abstract (precis)

Domestication led to profound changes in human culture. During this period, humans used breeding strategies to select for desirable traits in crops and livestock. These practices led to genetic and phenotypic changes that are trackable through archaeological and genomic records. Bacteria, yeasts, and molds also experienced domestication during the agricultural revolution, but the effects of domestication on microbes are poorly understood in comparison to plants and animals.

## COMMENTARY

The development of agriculture ∼12,000 years ago was one of the most significant innovations in human history. By exploiting the genetic and phenotypic variation in plants and animals, early farmers used selective breeding to continuously cross organisms with desired traits, eventually yielding crops with more food content (i.e., larger/more seeds and fruits) that were easier to harvest (e.g., loss of seed shattering in grains) and livestock that were less aggressive and more fertile (1). The impact of domestication and agriculture cannot be overstated given that it was a primary factor in the growth of the human population from 10 million to 7.7 billion in as few as 500 generations (1).

Domesticated plants and animals have garnered significant scientific interest because of their importance in human health and the economy at large, in understanding human history, and in offering a powerful evolutionary model system to study the effects of population genetic processes. A wealth of archaeological, molecular, and genetic evidence also suggests that domestication occurred in a diverse collection of bacteria, yeasts, and molds during the agricultural revolution (2, 3). The discovery of microbial fermentation likely accompanied the need to preserve food surpluses associated with the rise of agriculture. Consider that unrefrigerated raw milk can spoil within hours, while hard cheeses (produced through microbial fermentation) can remain unspoiled for days to months. Microbes also transform our food by making it more nutritious and by producing desirable consistencies and sensory properties.

For plant and animal domestication, humans used selective breeding to select for particular traits. However, it is less clear how humans domesticated microbes, since our use of food microbes predates their discovery by thousands of years. Genomic studies have been used to reconstruct the history of domestication of the yeast Saccharomyces cerevisiae (used to make beer, wine, bread, etc.) (4, 5), the sake and soy sauce mold Aspergillus oryzae (6), and the blue cheese mold Penicillium roqueforti (7). However, few studies have experimentally modeled microbial domestication. Experimental evolution of wild microbes offers a powerful tool to mimic the domestication environment and track the emergence of major genotypic and phenotypic shifts.

A new article by Bodinaku et al. demonstrates the power of experimental evolution for both theoretical and applied purposes (8). The researchers modeled the environment of bloomy rind cheese (e.g., Camembert, Brie, Coulommiers, Chaource, etc.), in which the outer side of the cheese is dominated by the filamentous mold Penicillium camemberti. This mold is pleasing to the eye (i.e., it is white and produces few spores) and nose (i.e., it produces compounds that contribute to the buttery texture, flavor, and aroma of soft bloomy rind cheeses) and is safe to eat (i.e., it does not produce harmful toxins). The researchers tested whether the laboratory cheese environment could transform the undesirable traits of wild *Penicillium* species (i.e., heavy pigmentation and production of spores, toxins, and undesirable odors) into traits more comparable to those of P. camemberti.

For a microbe, the food environment is extremely cozy; there is an abundance of high-quality nutrients, the environment is stable and predictable, and neighboring microbes are not generally antagonistic. The researchers altered two of these variables to understand their influence on the rate of phenotypic change. Lineages of wild *Penicillium* species were grown in a “normal-cheese” environment, in a “low-cheese” environment, or in a scenario that alternated between normal cheese and low cheese availability for each generation. The low-cheese scenario was used to manipulate nutrient availability, while the alternating scenario was used to manipulate the periods of “feast or famine” that can occur when bloomy rind cheese is traditionally aged. Additionally, in the normal-cheese environment, wild *Penicillium* species were grown alone or with a yeast and with two bacterial species that are common to bloomy rind cheeses given that a diverse community of microbes produces most traditionally fermented foods and that interactions between community members could influence the rate of phenotypic change. In each scenario, the wild *Penicillium* species were grown for a week and then serially transferred to fresh cheese media. At the end of each generation, colonies were scored either as “wild phenotype” or as “domesticated phenotype.” At the conclusion of eight generations, toxin production, volatile compound profiles, and gene expression profiles were compared between mutants with the domesticated phenotype and the ancestral lineage. These parameters allowed the research team to narrow the variables that might have driven phenotypic change in wild *Penicillium*.

A looming issue in microbial domestication centers on the emergence of domestication traits. For domesticated plants and animals, molecular clock analysis, trait measurements, radiocarbon dating of archaeological remains, and ancient DNA sequencing have all provided insight into the appearance of key traits and their underlying genetic basis. However, these tools are difficult to apply in microbial systems because generation time and reproductive strategy are often challenging to determine and because microbes degrade quickly and are not routinely part of the archaeological record (although there are a few notable exceptions [9, 10]). How can researches experimentally test the temporal order of trait emergence in domesticated microbes?

Using the cheese rind experimental evolution system, Bodinaku et al. demonstrated that traits desirable for cheesemaking rapidly emerged and conferred fitness advantages. After only eight generations of growth in the normal-cheese environment without an accompanying community of microbes, mutant colonies dominated in one of the wild *Penicillium* strains compared to the nearly undetectable presence of mutants in the low-cheese environment and differing levels in the alternating environment. This pattern was consistent across replicates, which suggests that selection, rather than drift, shaped the frequency of mutant phenotypes. In addition, both wild *Penicillium* strains produced higher frequencies of mutant colonies when grown alone than when grown in the presence of a microbial community. These results suggest a trade-off between reproductive output and competitiveness in the presence of cheese microbes.

The appearance of mutant phenotypes clearly suggests a phenotypic shift during experimental evolution in the cheese environment. However, whether these traits correspond to characteristics desired by humans in the cheese environment remained in question. To address this issue, researchers compared several key phenotypes between mutants with domesticated phenotypes and the ancestral lineage that had not undergone serial passage in the cheese environment. A functional hallmark of microbial domestication is the loss of secondary metabolism, which includes toxin production (2, 3). The loss of pigmentation was the first indicator that secondary metabolite production had been affected/lost in *Penicillium* mutants (see the bottom of Fig 3 in reference 8 for the clearest comparison). Pigmentation is primarily a consequence of the presence of melanin, a secondary metabolite. To test whether this was a widespread pattern or was exclusive to melanin, the research team performed further analyses. In addition to melanin, cyclopiazonic acid (a secondary metabolite that has toxic effects to mammals) either was not detectable or was greatly reduced in level in mutants with the domesticated phenotype. Gene expression profiles were also compared for one mutant with a domesticated phenotype and its ancestral lineage under conditions of growth in the laboratory cheese environment. This analysis revealed extensive downregulation of genes in the mutant lineage involved in secondary metabolism, including genes in the melanin- and ergot alkaloid-encoding gene clusters.

Defense is a central function of fungal secondary metabolism, be it to protect the cell from UV damage or to fend off competitors in an effort to protect a nutrient source. The fermented food environment exhibits greatly reduced biotic and abiotic stressors, and the loss of secondary metabolite production likely reflects a strategy to reallocate energy to primary metabolism and/or to preserve the optimal microbial community structure.

Mutant lineages of wild *Penicillium* may look domesticated (white) and act domesticated (reduced toxin production), but do they smell domesticated? To address this question, the researchers quantified and compared the aroma compounds produced by colonies with mutant and ancestral phenotypes. Amazingly, mutants with domesticated phenotypes did not produce geosmin, a compound with an earthy beet-like smell that is generally undesirable for bloomy rind cheese. The mutants with domesticated phenotypes did, however, produce substantially higher levels of several ketones that impart buttery, fatty, and cheesy aromas. This reminds us that if it looks like a cheese fungus, acts like a cheese fungus, and smells like a cheese fungus, then it probably is a cheese fungus.

The speed of these trait transformations is remarkable but perhaps not surprising. For instance, it took only six generations of selective breeding of the red fox for the emergence of docility (foxes that licked the hand of experimenters and wagged their tails when humans approached) (11). The rapid acquisition of desired traits may suggest that such traits in domesticated microbes are not necessarily discrete and sequential, as might be expected with organisms that primarily reproduce asexually. Instead, these traits could be the product of pleiotropic mutations or epigenetic modifications. For example, a single loss-of-function mutation in a global regulator of secondary metabolism could simultaneously disrupt production of toxins (e.g., cyclopiazonic acid and ergot alkaloid) and pigmentation-associated compounds (e.g., melanin) while freeing cellular energy for primary metabolism (e.g., ketone production).

Domestication implies phenotypic change driven by genetic change. The whole genomes of five mutants with domesticated phenotypes were sequenced and analyzed, but candidate mutations that could explain the domesticated phenotypes were not identified. This result implies that phenotypic variation has an epigenetic basis or that mutations are difficult to assess because of the annotation quality of the *Penicillium commune* reference genome. To eliminate epigenetic variation as a source of phenotypic plasticity, it would be helpful to test whether growing mutants in stressful environments could restore ancestral phenotypes.

Finally, the results from the study by Bodinaku hint at an exciting application of experimental evolution. Opportunities exist to harness the metabolic diversity of wild microbes to enhance the sensory properties of fermented foods. The rapid loss of toxicity during specialization in the food environment opens the door to safe discovery and screening of new volatile compounds that can be used to produce foods with new appealing flavors and aromas. For example, the usage of wild non-S. cerevisiae yeasts as bioflavoring agents in beer and wine is being explored (12). There is a vast, rich, and flavorful microbial world to explore, and experimental evolution offers a practical method to enhance food fermentation.
